# Voluntary Loss of Muscular Action

**Published:** 1847-12

**Authors:** 


*Voluntary Loss of Muscular Action.*—From the Christian Reflector we extract the annexed account of the long disuse of one the limbs of the human body. Many similar facts are on record, illustrative of physical endurance, and of the force of customs based on false views of religious duty :—

"It may seem incredible, but it is undoubtedly true, that there now exists at the Marmandilla Fank, in the middle of the city and island of Bombay, British India, a human being who has inhabited a summer-house, and held, on the palm of his left hand, a heavy flower-pot for twenty-one years without intermission. The narrator of this circumstance actually saw the hermit (for such he is called). The arm is completely sinew bound and shrivelled, the nails of his fingers are nine inches long, and curved like the talons of a bird. His beard nearly reaches to the ground when standing erect.

"While sitting, the man rests his elbow on his knee, and when walking he supports it with the other hand. His countenance indicates intelligence, and he once had very extensive possessions. All he now possesses is a few rags round the middle of his body, and a servant who is allowed to attend to his immediate wants, the pecuniary part of which is supplied by visiters.

"Twenty-one years ago he lost his caste by eating mutton ! an indulgĕnce in totally forbidden food, and was consequently condemned to hold, for 30 years, a large flower-pot filled with earth, in which grows a sacred plant. To lose caste, and not be able to take it up again, according to the superstitions of these deluded idolators, is to incur the penalty of everlasting misery in a future state. What an example does this poor deluded creature afford of perseverance, zeal, courage and devotion, worthy even of the highest cause. If he live to redeem his caste, most likely he will hereafter be set apart to be worshipped as a god."—*Ibid.*

